# An open-source dual-beam spectrophotometer for citizen-science-based water quality monitoring

**DOI:** 10.1016/j.ohx.2021.e00241

**Published:** 2021-10-19

**Authors:** Jiansheng Feng, Banafsheh Khakipoor, Jacob May, Melissa Mulford, Joshua Davis, Kelly Siman, Gabrielle Russell, Adam W. Smith, Hunter King

**Affiliations:** a Department of Polymer Science, University of Akron, Akron, OH, USA; b Department of Biology/Integrated Bioscience, University of Akron, Akron, OH, USA; c Department of Chemistry, University of Akron, Akron, OH, USA

**Keywords:** Open source hardware and software, Water quality monitoring

## Abstract

Efforts to understand and mediate threats to water supplies rely on collection of reliable data at large scale, a goal which is often limited by availability of tools that are both affordable and reliable. We present here a low-cost, easy-to-use, do-it-yourself (DIY) spectrometer for measurement of a variety of relevant solute concentrations when coupled with inexpensive commercially-available reagents. Comparison of its performance with commercial options demonstrates the potential value of this device as transparent, versatile, and accurate-enough alternative for widespread application.

## Graphical abstract

**Figure:**
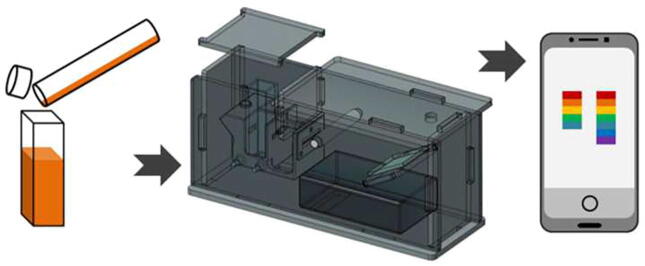

**Specifications table:**

**Table:** 

**Hardware name**	*Erie Open Spectrometer (EOSpec)*
**Subject area**	
	•*Chemistry*
	•*Biological Sciences*
	•*Environmental, Agricultural Sciences*
	•*Educational Tools and Open Source Alternatives to Existing Infrastructure*
	•*General*
**Hardware type**	
	•*Imaging tools*
	•*Field measurements*
**Open source license**	*MIT License*
**Cost of hardware**	$*30*
**Source file repository**	https://doi.org/10.17605/OSF.IO/Q2BPN

## Device in Context

Water resources worldwide are experiencing increasing stresses from factors related to the compounding effects of climate change and population growth. This, in turn, has numerous profound ramifications at the global scale. Industrial and agricultural runoffs represent a subset of problems by which even relatively low concentrations of artificial pollutants threaten the water security of local communities. Two specific cases, agricultural runoff and acid mine drainage, exemplify the need for inexpensive water quality measuring tools for field use by citizen scientists.

All around the world, agricultural nutrient runoff is the main cause of harmful algal blooms (HABs) in lakes and red tides in oceans. These overgrowth of otherwise benign cyanobacteria can poison drinking water, overwhelm even sophisticated water treatment facilities [1], and devastate wildlife and aquaculture [2], [3].

Gold and coal mining are major contributors of acid mine drainage. Ores containing gold, copper, and various other desirable metals are rich with iron sulfide. Extracting metals form these rocks involves crushing these ores, which exposes iron sulfide (Iron(II) disulfide, ${\mathrm{FeS}}_{2}$) to oxygen in air or water, sequestering various pollutants including sulfur dioxide and sulfuric acid [4]. Acid mine drainage, once started, can continue releasing iron sulfide for hundreds of years [5].

While, in either case, changing the practices upstream might not be an easily available solution, there is tremendous value in simply knowing the levels of contaminant downstream. The ability to either predict HABs or to evaluate the resilience of aquatic ecosystems requires nutrient loading data with high spatial and temporal resolution along the watershed [6]. Knowing the Fe(II) and sulfide levels in a river can help research, monitoring, and rehabilitation efforts, or prevent expensive industrial failures [7]. The interest of mining firms in developing countries, has created social changes where communities are divided between accepting/supporting versus confronting/grappling with mining activities [8]. In some cases, government agencies and coal industry fail to provide transparent monitoring record, resulting in mistrust. These can be addressed by enabling a large number of residents to participate in monitoring their environment [9].

While microscopy and light scattering techniques can be used to quantify the number density and the size distribution of particulate matters suspended in water, measurements of chemical or elemental concentrations are often achieved by various analytical chemistry methods such as gravimetric analysis and titration. In some cases, a solute’s interaction with light can be analyzed for chemical identification and quantification of concentration. When light is passed through a solution, molecules in the solution absorb a portion of the light according to their available electronic transition energies. This process produces a characteristic attenuation of transmitted light intensity as function of wavelength. For a given wavelength, intensity *I* transmitted through a solution can be related to molecular concentration based on the Beer–Lambert law:

(1) $$I(c)=I_{0}e^{-∊\mathrm{cl}}$$

where $I_{0}$ is the incident intensity, *c* the concentration, *l* is the optical path length, and ∊ the molar attenuation coefficient, which is a function of wavelength. In other words, the Beer–Lambert law states that the absorbance, $A=-\ln(I/I_{0})$, is proportional to the solute concentration, c. This elegant law relating concentration to light intensity is the basis for many practical methods for monitoring water quality.

In a spectrometer, light is passed through the sample and subsequently a dispersing element, generating the corresponding spectrum across a light sensor array, allowing for, in principle, measurements of absorbance at any wavelength of interest. By contrast, colorimeters, lacking a dispersing element, measure absorbance of a specific wavelength in visible spectrum [10].

With a single-beam spectrophotometer, $I_{0}$ and *I* can be measured in successive images, before and after a sample is inserted, and reliable absorbance measurement can be obtained only if lighting conditions and sensor settings are unchanged. A dual-beam device compares, in a single image, two spectra resulting from two beams - one passing through a reference ($I_{0}$) and the other through a sample solution (*I*) This strategy makes the measurements more robust, because it has effectively eliminated the errors caused by the variations in lighting conditions and sensing parameters [11]. Here we have further developed the dual-beam spectrophotometer by incorporating a built-in light source. This revamp not only enhances the device’s field-readiness, but also, more importantly, improves its measurement repeatability. A built-in light source can help minimizes two sources of systematic error: (i) the variation in the relative incident-light intensity of the two beams, and (ii) the variation in peak locations (in wavelength) of the incident spectrum.

As long as each pixel reports a signal proportional to light intensity, the ratio $I/I_{0}$ can be evaluated despite unknown parameters (e.g. white-balance settings of a third-party sensor). To quantify concentration, a wavelength is chosen for which ∊ is sufficiently large. When using a DIY spectrometer, a calibration curve is first generated with standard samples and verified using the Beer–Lambert law. Once the calibration is established, it can then be used to calculate concentrations of unknown samples from their absorbance.

In principle, the absorbance spectrum of an untreated water sample should reveal the presence and quantity of its contaminants. However, in practice, the absorption signal from the solutes of interest at environmentally relevant concentrations are typically either outside of the visible spectrum or well below the sensitivity level of affordable and commonly available measurement devices. A practical solution to this problem is to use chemical reagents, which upon reacting with the solute produce a large absorbance signal in the visible spectrum range. Such reagents are commercially available for a variety of chemicals of interest, and are integral to both color-strip tests [12] and spectrometer-based, EPA-approved protocols for water quality measurement [13]. For a color-strip measurement, the resultant solution is to be compared to a printed reference card, and a human user has to make a judgment on where its tone would land on the color scale. In either case, the accuracy of the analytical measurement is limited by both the performance of the reagent and the uncertainty in quantifying the optical signal.

Water quality is most commonly measured using commercial devices [13], but due to their cost, often a very small number of devices need to be shared between many citizen scientists, and that significantly limits the amount of measurements. Currently, scientists either use expensive field tools or take samples back to labs for these measurements. For local communities to be able to take part in monitoring their own water, they must be equipped with effective tools. Also, for that participation to lead to a meaningful broader impact, the measurement process needs to be straightforward without sacrificing the accuracy of the results.

Existing options for monitoring water quality at scale are neither prohibitively expensive nor difficult to find. Their designs range from black boxes which promise accuracy but little engagement or transparency, to cheaper, reactive paper, which rely on subjective judgment of color tone. None seems to have been designed to engage the user in the process of inquiry or reveal its underlying logic. Moreover, none was specifically designed to engage the user with the existing data set to which they are contributing, a key part of the role as citizen scientist. Development of the device described below followed from these needs.

## Hardware description

Currently, several commercial spectrometers are available for water quality measurements. However, they are usually either: relatively expensive for individual use, starting at ~$200 [13], [14], [15], [16], [17]; are hard-wired to specific wavelength for one or two particular chemicals [18]; or incorporate black-box design and are difficult to troubleshoot for non-technical users [13], [18], [14], [15], [16], [17].

For the sake of large scale implementation, we aim for a pedagogical, effective, minimally sophisticated alternative, along the lines of DIY and classroom solutions (excellently summarized by Ref. [19]), to be specifically:

• Affordable and accessible for researchers and citizen scientists.
• Reliably accurate for the needs of large scale field monitoring.
• Adaptable to various measurements with appropriate calibration.

**Fig. 1:**
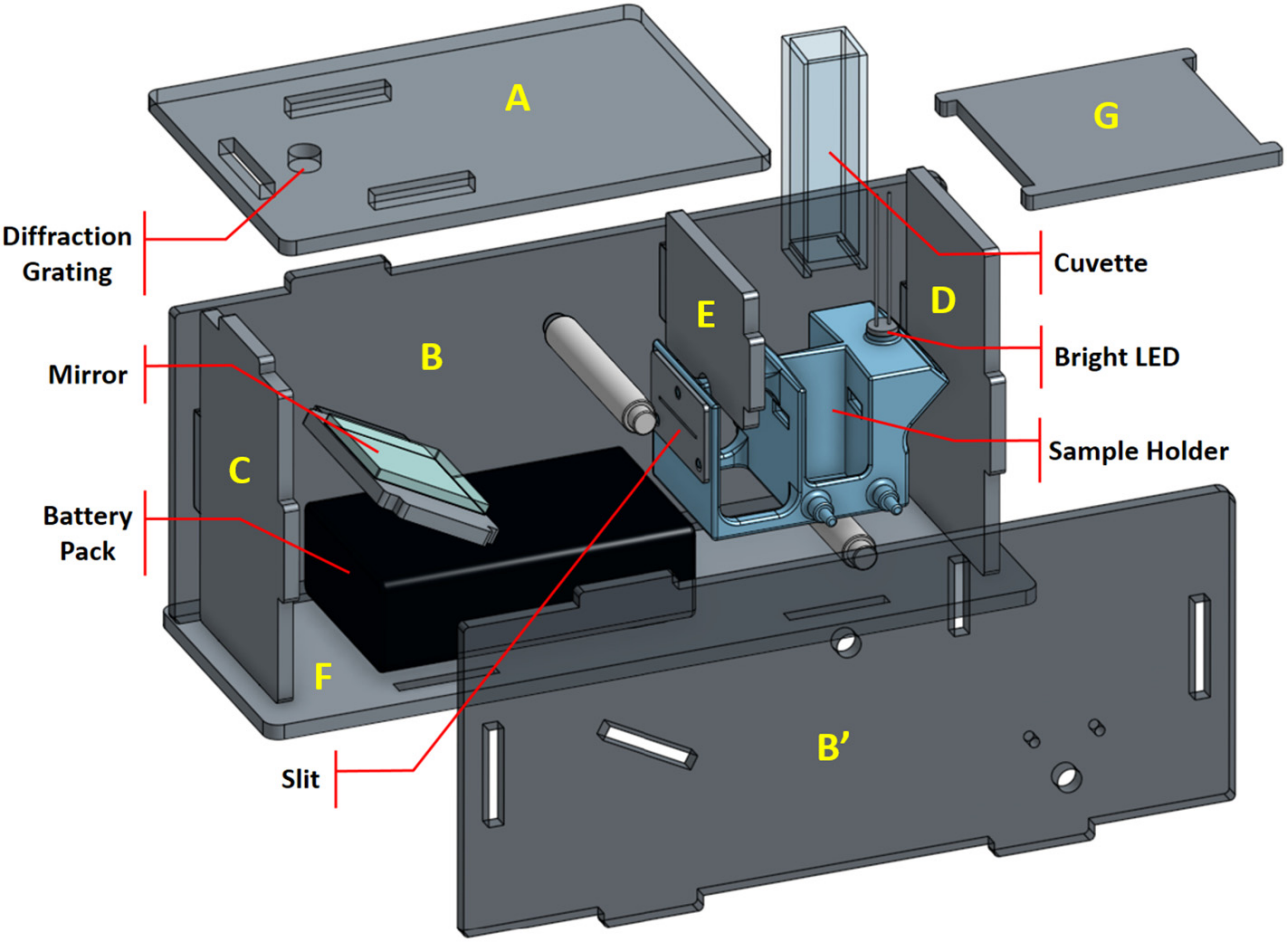
Exploded view of EOSpec showing the components. Enclosure panels are labeled for reference in build instructions section below.

We describe here a visible-spectrum spectrophotometer for large-scale citizen-science water-quality measurement, called EOSpec (pictured in Fig. 1). It uses exclusively low-cost, easily available materials, and requires only fab-lab tools commonly available in many public libraries and schools (3D printer, laser cutter). The hardware implements an internal dual beam light source and dispersing element to create an image on the sensor of the user’s own smartphone. Quantitative analysis of the resulting image can then extract absorbance as function of wavelength, and subsequently solute concentration if a calibration table is available. Data presented in the validation section was collected using a custom-built smartphone app. This option is useful for rapid evaluation and data sharing possibilities.

Flexibility and transparency of the design contributes to the stated goals in two ways. First, through open access and guided tutorials, users are encouraged to learn the science behind spectrometry, which is useful for educators and troubleshooting, and contribute to development. Second, any piece of the device can be repaired or replaced as needed if damaged or needed upgrades, without requiring a new device. The device can be calibrated for most visible wavelengths to return an absorbance value, allowing for large scale use by citizen scientists to measure various compounds of interest in the field.

## Design files

**Table:** 

**Design filename**	**File type**	**License**	**Location of the file**
EOSpec enclosure	DXF file	Creative Commons - Attribution - Non-Commercial - Share Alike	https://doi.org/10.17605/OSF.IO/Q2BPN
EOSpec sample holder	STL files	Creative Commons - Attribution - Non-Commercial - Share Alike	https://doi.org/10.17605/OSF.IO/Q2BPN
EOSpec slit 0.5 mm	STL files	Creative Commons - Attribution - Non-Commercial - Share Alike	https://doi.org/10.17605/OSF.IO/Q2BPN
Code: algorithm explanation	iPython Notebook	MIT license	https://doi.org/10.17605/OSF.IO/Q2BPN
Code: image analysis, minimal	Python	MIT license	https://doi.org/10.17605/OSF.IO/Q2BPN
Code: image analysis, with alignment function	Python	MIT license	https://doi.org/10.17605/OSF.IO/Q2BPN
iOS UI	Xamarin	MIT license	https://doi.org/10.17605/OSF.IO/Q2BPN
iOS main view design controller	Xamarin	MIT license	https://doi.org/10.17605/OSF.IO/Q2BPN
iOS main view logic code	Xamarin	MIT license	https://doi.org/10.17605/OSF.IO/Q2BPN
iOS result page design controller	Xamarin	MIT license	https://doi.org/10.17605/OSF.IO/Q2BPN
iOS result page logic code	Xamarin	MIT license	https://doi.org/10.17605/OSF.IO/Q2BPN

• EOSpec sample holder (Thingiverse) positions inside the cut patterns and holds reference and sample cuvettes. User needs a resin 3D printer to print EOS sample holder. Extruder 3D printers could work but need a level of experiments to get the best outcome.
• EOSpec slit (Thingiverse) is added to the sample holder to restrict the light passing through it which necessitates the use of resin 3D printers due to their higher level of accuracy.
• EOSpec enclosure (Thingiverse) serves as structural support and light enclosure component of the spectrometer. It contains nine pieces that can be printed using a laser cutter. The pieces are designed to fit into each other similar to LEGO bricks. However, depending on laser cutter used, some pieces might need an appropriate amount of force to fit in.
• Code was written in Python (GitHub), and finds spectra in an image and uses the RGB values between reference and sample spectrum to quantify the transmitted intensity over specific wavelength range and calculates absorbance which can be calibrated to concentration of specific nutrient in water sample.
• iOS application was developed using Xamarin/Visual Studio. It takes the picture produced by spectrometer, and analyzes it similar to Python code and return an absorbance. This option trades some opacity in the details of algorithm for ease of use in the field. The application also offers a ‘blank’ option to calibrating device at each run using blank samples.

## Bill of materials

Bill of materials (BOM) is divided in two sections. Table 1 shows the hardware cost and Table 2 shows the cost for two example reagent kit plus accessories needed to perform the tests. Materials in both tables are needed to perform testing described in this paper.

**Table 1:** All the hardware pieces and their estimated costs for one unit. Many materials are not sold in pieces and hence the unit tests are estimated when individual sale is not available.

**Component**	**Source**	**Purchase Price**	**Quantity Needed per Unit**	**Cost per Unit**
resin (standard)	Formlabs	$149 per 1 liter	16.3 mL	$2.5
isopropyl alcohol	Amazon	$22 per 950 mL	50 mL *	$1.5
acrylic sheet (1/8”-thick)	McMaster-Carr	$7.2 per 12”×12” sheet	1	$7.2
diffraction grating	Amazon	$13 per 10	1	$1.3
mirror	Michaels	$2 per 25	1	$0.1
battery pack	Adafruit	$2.0 each	1	$2.0
LED	Adafruit	$7.0 per 25	1	$0.3
resistor	Adafruit	$0.8 per 25	1	$0.1
spacer	McMaster-Carr	$2.15 each	2	$5.4
screws	McMaster-Carr	$8 per 100	4	$0.3
hex nuts	McMaster-Carr	$4 per 100	4	$0.2
Teflon sheet 1/32”-thick	McMaster-Carr	$4.4 per 6”×6” sheet	2”×2”	$0.5

**Table 2:** Many reagents test kits can be purchased online, Nitrate and phosphate are two example of the reagents costs. pipettes and plastic vials are for collecting samples and mixing reagents with samples.

**Component**	**Source**	**Cost**	**number of tests**	**Cost per test**
API nitrate test kit	Amazon	$11	90 to 180	$0.1
API phosphate test kit	Amazon	$14	90 to 180	$0.1
3 mL pipette (110 pack)	Amazon	$10	110	$0.1
5 mL plastic vial (100 pack)	Amazon	$10	100	$0.1
3.5 mL cuvette (100 pack)	Amazon	$10	100	$0.1

## Build Instructions

### Spectrometer Hardware

Step 1: Sample holder and slit.

**Fig. 2:**
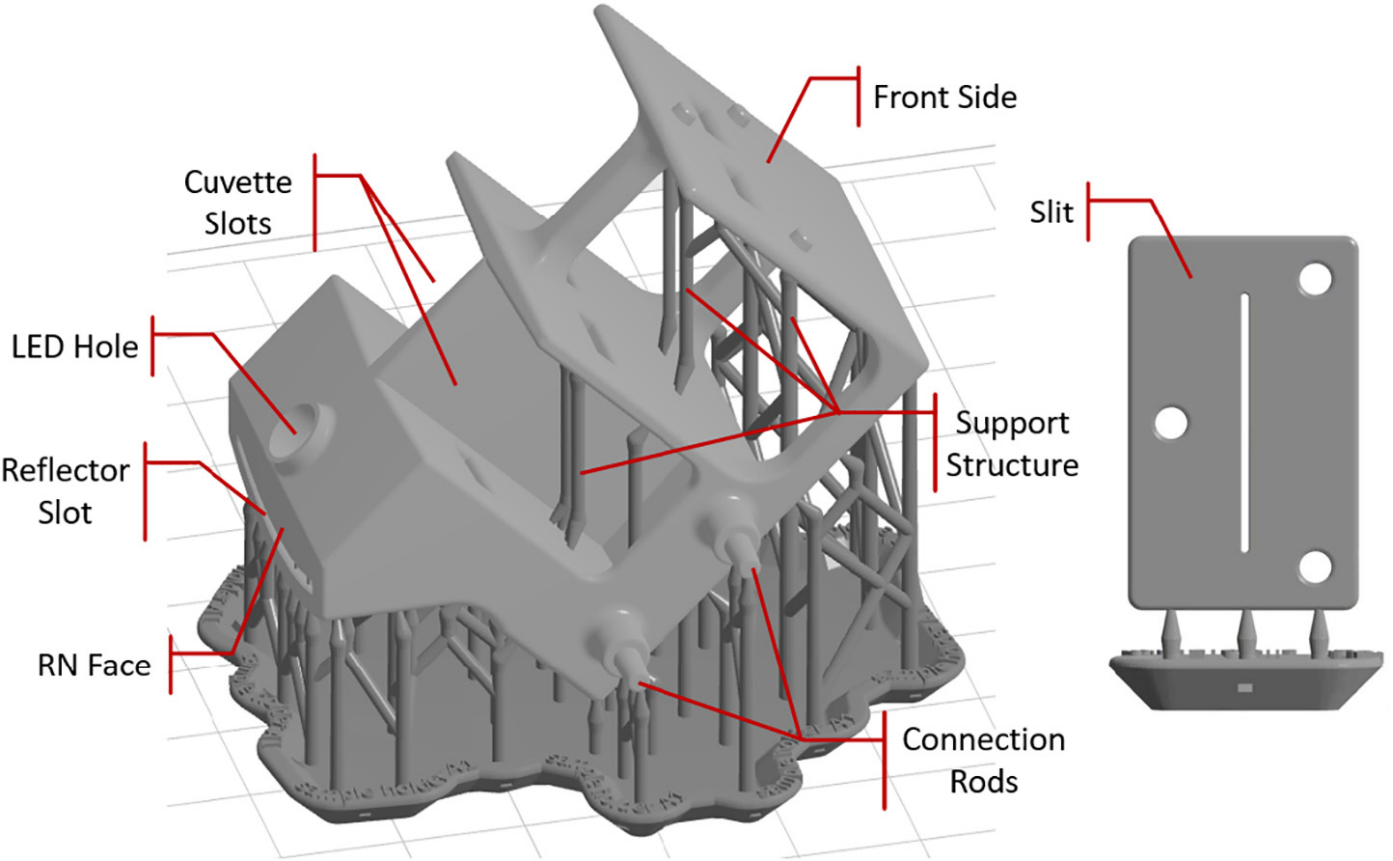
Sample holder (left) and slit (right) are 3D-printed by an SLA printer to ensure high geometric fidelity. The support structures necessary to achieve a successful 3D-print are shown in dark gray. They need to be removed prior to assembly.

Sample holder and slit (Fig. 2) are 3D-printed with a commercial desktop SLA printer (Formlabs Form 2). SLA (stereolithography) type 3D-printers are recommended over FDM (Fused Deposition Modeling) type, mainly because of their superior accuracy which is needed to ensure the geometric fidelity of the printed parts. The open-source 3D-design (STL) files are shared on osf.io (refer to Design Files section). After the design files are loaded onto the 3D-printer control software (Formlabs Preform), a few final preparation steps are required including orienting the parts and generating the support structures. It is found that the orientation of the parts are of significant importance to achieving successful prints. As shown in Fig. 2, the sample holder should be oriented with the surface labeled ”RN face” (reflector-normal face) on the upright plane, otherwise the reflector slot may not form properly. The slit should also be oriented on the upright plane with the slit opening pointing vertically in order to obtain the best printing result. The density of supports and the touchpoint size are also found to affect the quality of the print to various degrees. It is recommended to increase support density by 20% from the default value and decrease touchpoint size to 0.5 mm.

The sample holder has placement slots intended for reference and sample cuvettes. They are designed for standard 5 mL cuvettes (10 mm×10 mm×50 mm nominal) to fit snugly. It is worth noting that there are support structures inside those slots, and they need to be removed prior to assembly. Artist knifes or exacto knifes have been found to be suitable for used to remove the supports and smooth the inner surfaces. It can be noticed that the orientations of the two cuvette slots are converging at a slight angle ($\approx 7^{o}$). This is so that the two light beams, each passing through the respective cuvette perpendicularly, converge at the diffraction grating, making sure the two spectra appear side-by-side on the cellphone camera. (Note: The directionality of the light beams is enforced by the three pairs of windows - one on the back side of the cuvette slot, one on the front side of the cuvette slot, and one on the front side of the sample holder. In other words, the only light beams that can get to the diffraction grating are the ones that are perpendicular to the sidewalls of the cuvettes.).

The connection rods coupling the sample holder to the enclosure (more details in Step 4) are slightly conical (with tip slightly smaller than base) for a snug fitting. The touchpoints on them may also need to be smoothed out.

Step 2: Installation of reflector, LED, and slit.

**Fig. 3:**
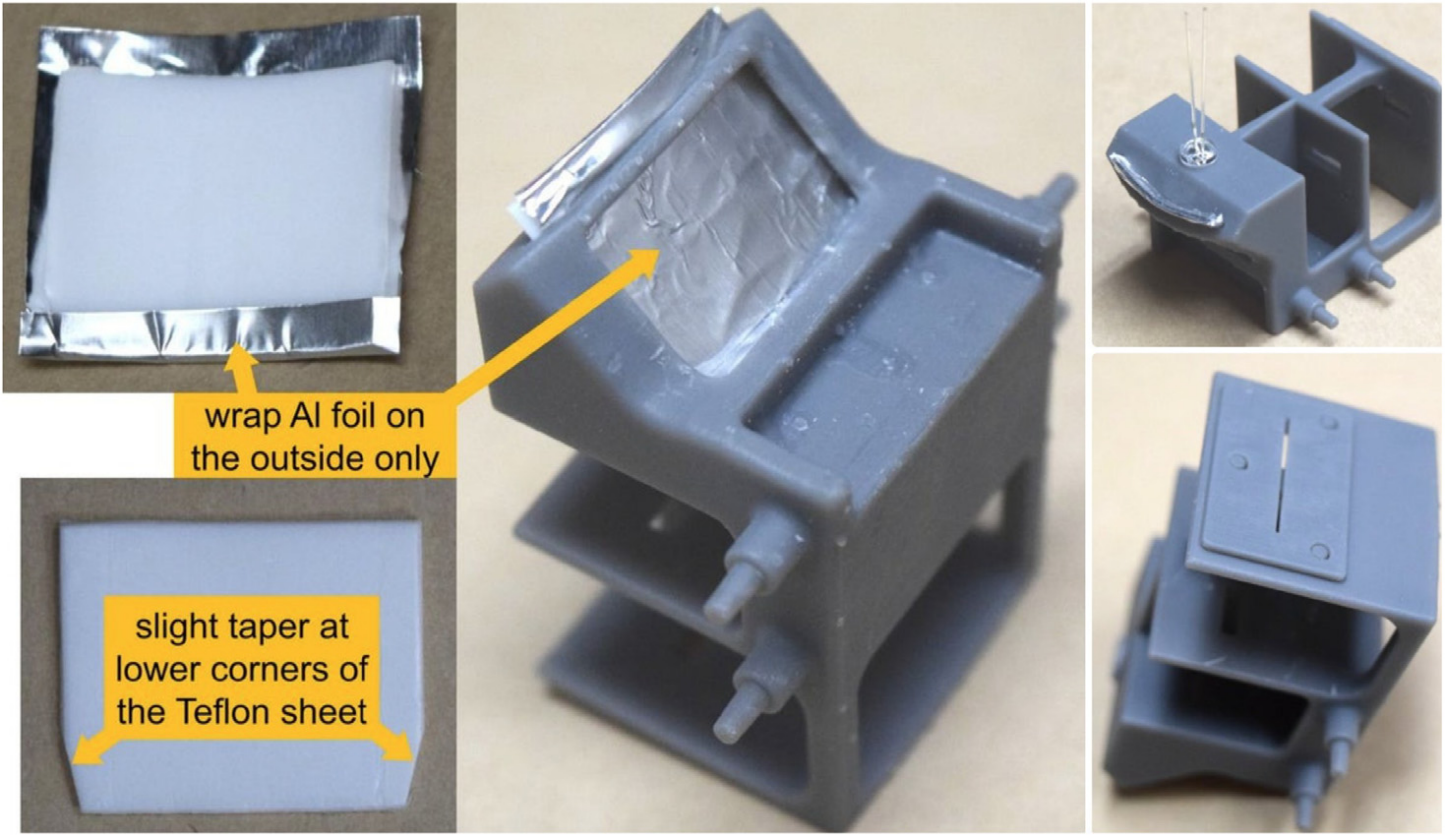
A Teflon sheet, with an optional aluminum foil, is used as reflector (left). The reflector is inserted in the reflector slot, which is a curved gap at the backside of the sample holder (middle). The LED is installed inside the LED hole, and the slit is attached to the front face of the sample holder (right). This arrangement helps to improve the uniformity across the width of the spectra.

A piece of white Teflon (PTFE) sheet (1/32”-thick), chosen for its flexibility, color, and reflective characteristics, is cut into a roughly 2”×2” piece to be used as a reflector. A piece of household Aluminum foil may be added to the back side of the Teflon sheet to enhance reflectivity. The reflector (Teflon with Al foil) is then inserted into the slightly curved slot at the back of the sample holder. Careful iterative trimming is often required for successful insertion. Slight tapering at the leading corners can help with insertion. A bright white LED inserted into the hole near the reflector slot is used as the light source (Fig. 3). Depending on the quality of construction, the incident-light intensity going to the two cuvettes may have small but measurable difference. This difference will be automatically accounted for during the calibration process, because the ratio of the two intensities stays constant over time.

The slit is 3D-printed separately to allow for better control over the quality of the print. The purpose of the slit is to provide a very narrow light beam (≈0.5mm), which helps to improve spectral resolution of the signal. Although spectra can still be obtained without the installing the slit, more color mixing will occur, and the 2nd-order spectra can also appear in the photo (more detail in Operation Instructions section), which may lead to software execution errors caused by mis-identification of the spectra. To be clear: the slit is not used as a dispersion element, but rather to trim the width of the light beam to improve spectral resolution. (Dispersion via diffraction grating will be discussed in Step 5.) The slit is anchored by three connection posts and attached to the front face of the sample holder with double-sided tape or superglue.

Step 3: Enclosure panels.

**Fig. 4:**
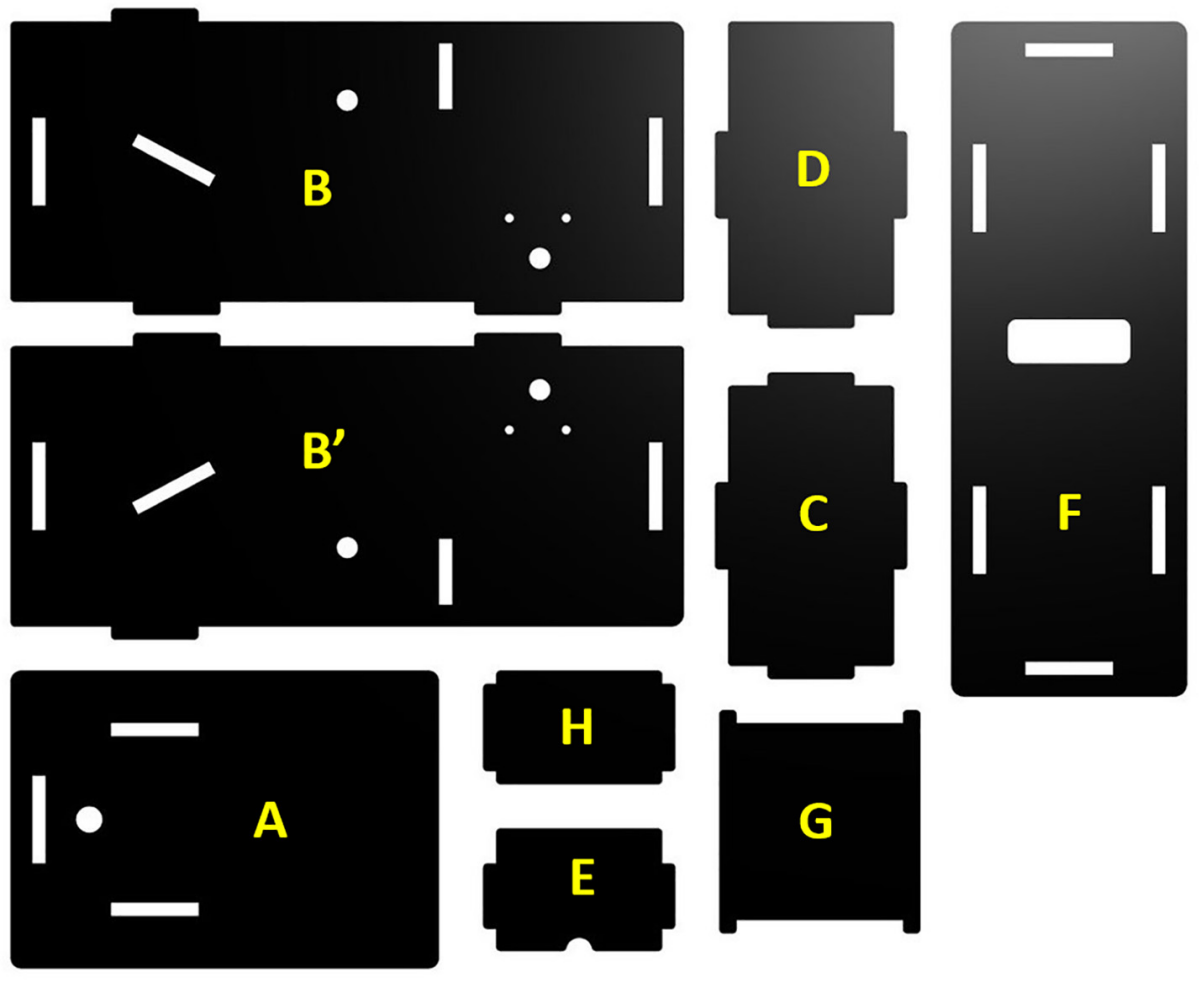
Laser-cut acrylic panels are used to form the enclosure. Panel labels correspond to those shown in Fig. 1. Notice (H) is the panel on which the mirror is attached, and it was not labeled in Fig. 1 to avoid confusion with other components i.n its proximity.

The hardware enclosure is constructed from panels that fit together like a puzzle box (Fig. 1) and held together by two sets of screws. As shown in Fig. 4, the panels are cut out from an opaque 1/8”-thick 12” × 12” acrylic sheet using a commercial laser engraver (Epilog, 40 W). The open-source 2D-design (DXF) files are shared on osf.io (refer to Design File section). Clear acrylic sheets should be avoided because an important function of the enclosure is to block out ambient light. PVC sheets should also be avoided because, when heated, PVC produces hydrochloric acid which is not only a health hazard but also corrodes the laser cutting equipment.

The acrylic sheets purchased from McMaster-Carr (refer to bill of materials) come with protective cover papers on both sides. It is recommended that they are kept in place throughout the entire construction process, and that only the outside cover paper be removed after the EOSpec is assembled. Keeping the inner cover paper can help reduce measurement noise by (i) minimizing the small amount of ambient light penetrating through the enclosure and (ii) reducing the specular reflection of the built-in light source on the inside surfaces.

Step 4: Enclosure assembly.

**Fig. 5:**
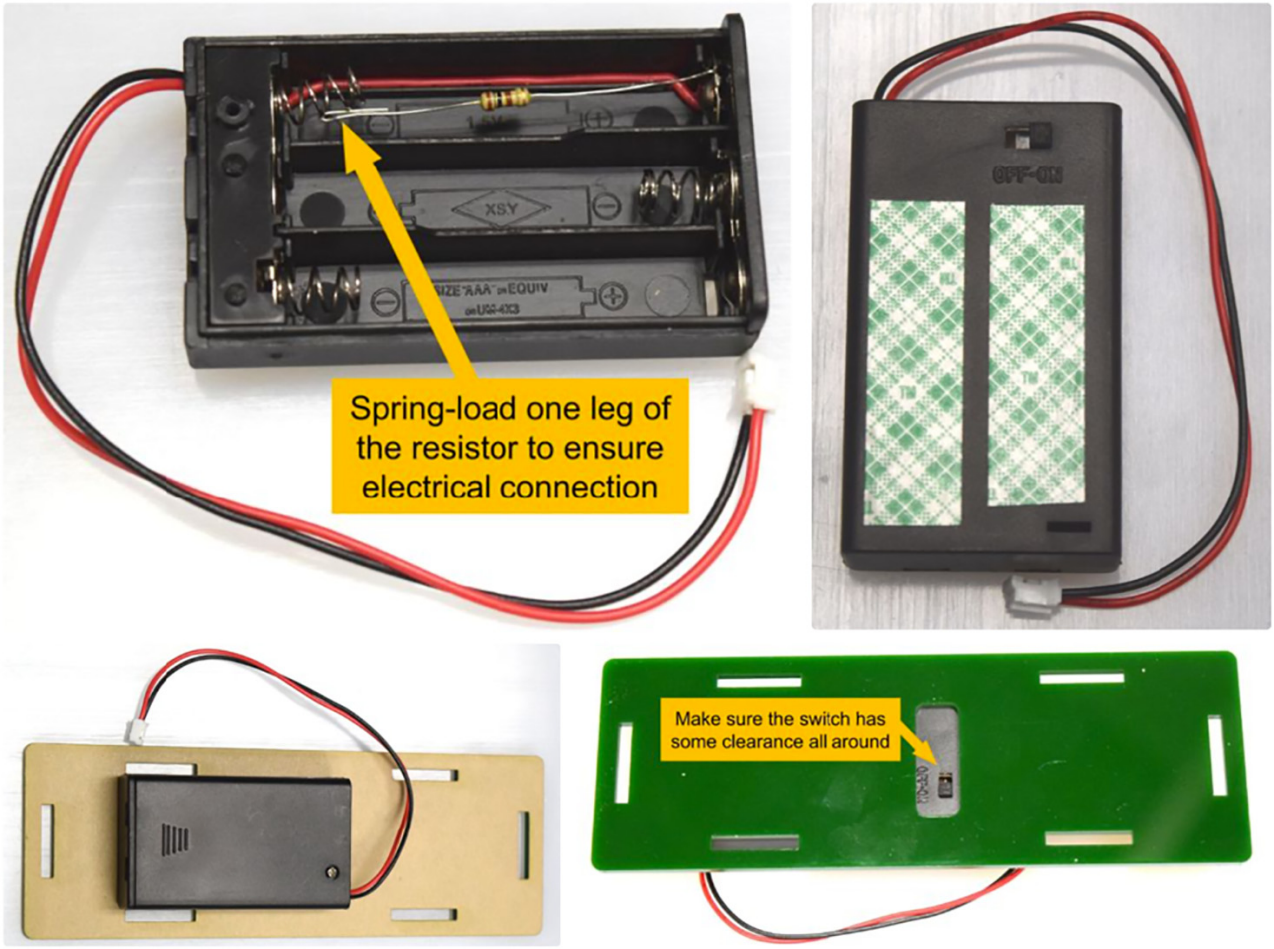
A 100Ω resister is installed in one of the slots inside the battery pack, while the other two slot each hold an AA battery (not shown). Double-sided tape is used to attach the battery package to bottom panel with the switch accessible through the opening.

The battery pack needs to be slightly modified by adding a current-limiting resistor prior to device assembly. As shown in Fig. 5, a 100 Ω resistor is spring-loaded into one of the slots inside the battery pack. Because the three slots in the battery pack are connected in series, it does not matter which slot the resistor is installed. After installing two AAA batteries into the two remaining empty slots, the battery pack is secured onto panel F in Fig. 4 with double-sided tape The battery pack should be positioned such that the switch is accessible from the outside through the cutout whole in the acrylic panel. An 1” squared mirror is attached to panel H in Fig. 4 with superglue or double-sided tape. Although the alignment of the mirror is not important, it is suggested that the mirror be centered at the panel with its edges roughly align with the edges of the panel.

**Fig. 6:**
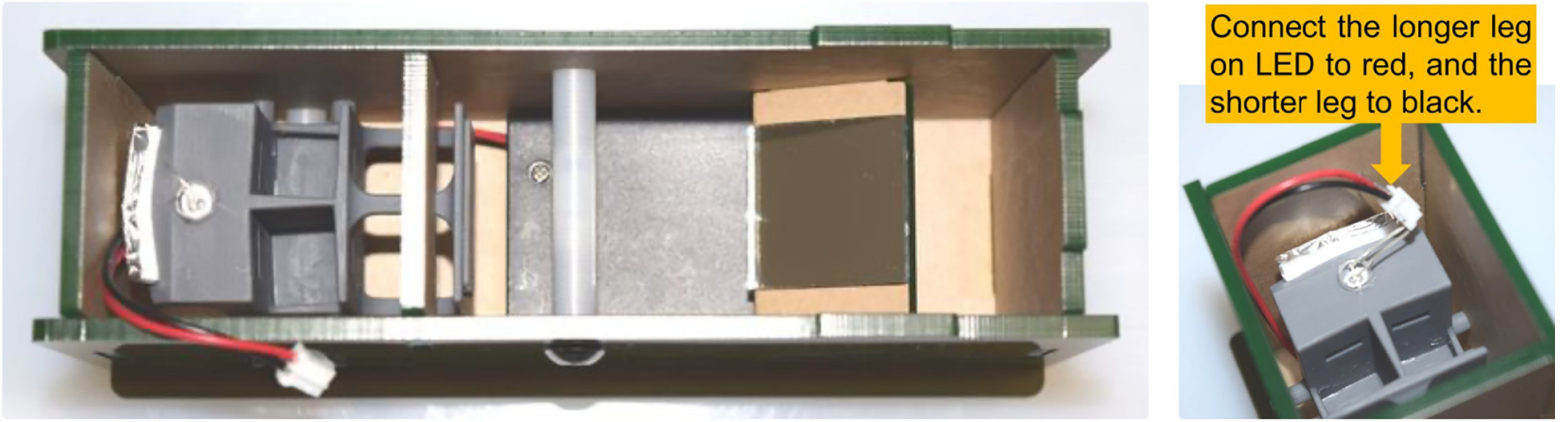
Wires from the battery pack are passed around the back side of the sample holder and connected to the LED. Caution is needed to ensure the correct polarity: red wire (positive) to the longer leg of the LED; black wire (negative) to the short leg.

The mirror panel, the front (C), the back (D), the blocker (E), the spacers, and the sample holder are then placed on a side panel (B) as shown in Fig. 1. After verifying the position and orientation of the parts are correct, the opposite side panel (B’) is then installed and secured with screws. Notice the two spacers are threaded and will take two screws each (one on either side). Before installing the bottom panel, pass the cable on the battery pack around the backside of the sample holder, as shown in Fig. 6, then connect wires to the LED. Make sure the red wire is connected to the long leg on the LED, and the black wire to the short leg.

Step 5: Diffraction grating installation.

**Fig. 7:**
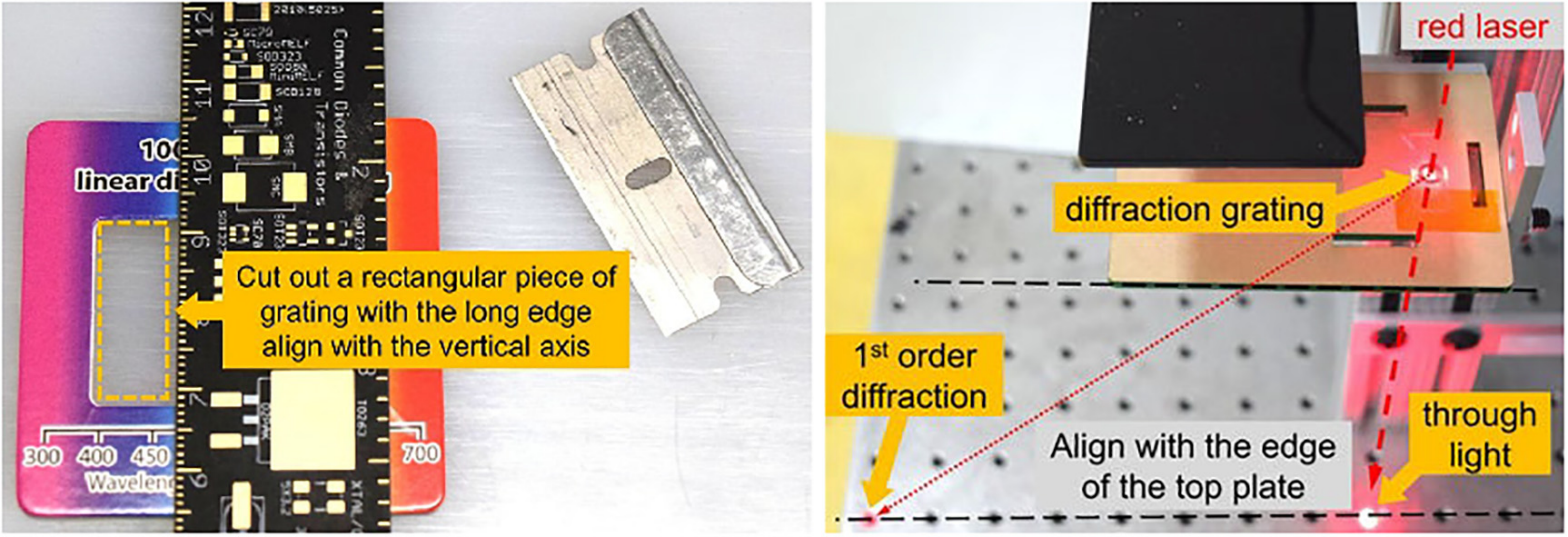
Aligning diffraction grating using a red laser light beam, where we pass red laser through diffraction grating hole and confirm first and second order resulting dots to be on the same line.

The dispersion element in the spectrometer is a commercially available educational grade diffraction grating (1000 lines/mm or better is recommended, refer to Bill of Materials). As shown in Fig. 7 (left), a small piece (≈ 1 cm × 2 cm) of transparent film is cut out from the cardboard frame with the long edge align to the vertical axis. The film is then placed onto the bottom side (i.e., the side facing inward when assembled) of the top panel (panel A in Fig. 4) over the observation hole. Special attention should be paid to ensure the long edge of the film is aligned with the short edge of the panel. A simple rig with a laser pointer (see Fig. 7, right) may be used to check the alignment. The straight line connecting the through light spot and the 1st order diffraction spot is the direction along which the spectra will be displayed, and it should be aligned with the long edge of the panel. Slowly rotate the grating film along the axis normal to the panel until satisfactory alignment is achieved. The grating film is then secured to the panel with Scotch tape.

**Fig. 8:**
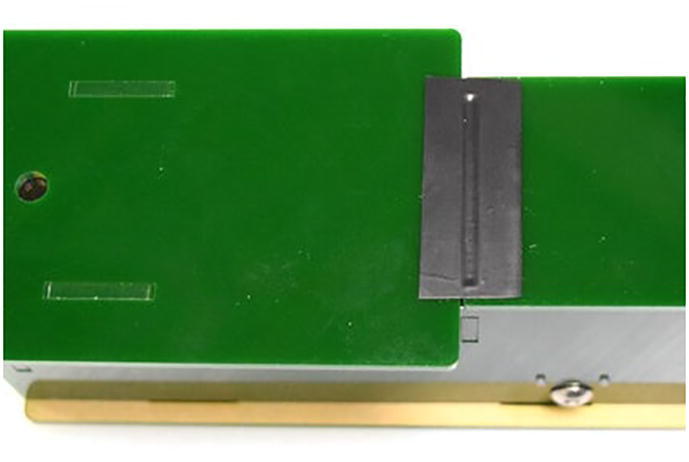
Electronic black tape is used as a hinge between the top and bottom cover of EOSpec.

Finally, the top panel is simply fitted onto the already-assembled device. Panel G in Fig. 4 is used as a cover over the sample compartment. A piece of electrical tape may be used as hinge (Fig. 8).

### Image analysis software

With the help of the spectrometer, a digital image containing two spectra (corresponding to the reference and the sample, respectively) can be captured with a smart phone (see Operation Instructions section for more details). The absorbance can then be calculated by analyzing the image. This can be done in a number of ways. We detail here a method shared as iPython Notebook, and present results obtained by the equivalent method implemented directly in a smartphone app, the EOSpec application), with a simplified user interface.

The input is expected to be an RGB image of two spectra side by side. Regardless of the input datatype, the primary data matrix is first converted to 32-bit floating point number format, where each channel is normalized to between 0.0 and 1.0.

In order to provide tolerance to imperfect positioning of the smart phone camera when taking the spectra image, accurately locating the spectra should be automated with limited assumptions of the input signal. Using a simple metric:

|R-G|+|R-B|+|G-B|

the vibrancy of each pixel in the image can be estimated. This vibrancy metric assigns high values to pixels with bright colors and zeros to pixels on the gray scale, and thus can effectively estimate the likelihood of a pixel being in a spectrum. For example, a bright red (R=1.0,G=B=0.0) pixel will be assigned a vibrancy value of 2.0, and so will bright green (R=B=0,G=1.0), bright yellow (R=G=1.0,B=0.0), etc. Any pixel with color on the gray scale (i.e., R=G=B), from pure black (R=G=B=0.0) to pure white (R=G=B=1.0), will be assigned a vibrancy value of 0.0. Therefore, the two colorful spectra can be sharply distinguished from the dark background as clusters of pixels with high vibrancy values. The pixel locations of the two spectra can then be identified by collapsing the two axes - one at a time - and applying thresholds corresponding to 20% of the peak value (Fig. 9, middle). After the spectra are located, the corresponding wavelength of each pixel is calculated based on the pixel locations of the red and the blue peaks on the reference spectrum (Fig. 9, right). The actual spectral locations of the peaks are measured by a commercial spectrometer, and since they are primarily determined by the light source (i.e., emission spectrum of the LED), those values can be applicable to any spectrometer using the same type of LED as light source. (Dependence of LED emission on varying battery voltage, sensitivity on reflector positioning, and the effect of camera sensitivity across the spectrum remain to be rigorously quantified.) In the EOSpec application, we have also fixed the camera settings (focus, exposure, ISO) to prevent them from introducing additional variability which may undermine measurement accuracy. Absorbance at a targeted wavelength can then be calculated by integrating the RGB values (or only the prominent channel) over the vicinity (e.g., ±5nm) of that wavelength, and compare the results between the sample spectrum and the reference spectrum.

**Fig. 9:**
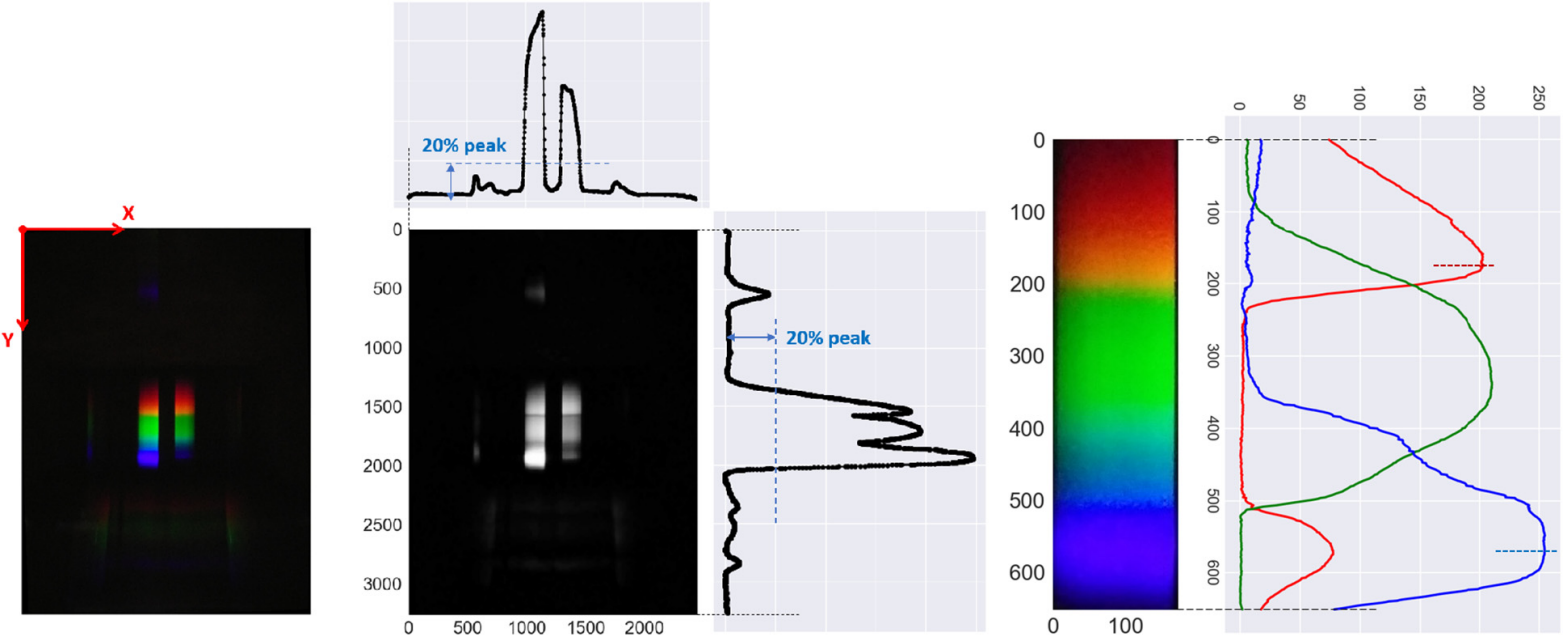
(Left) Spectrum image taken with a smart phone. (Middle) The corresponding vibrancy map where the two spectra, due to they high vibrancy values, are highlighted. The pixel locations of the spectra are identified by a 20% threshold. (Right) The resulting reference spectrum. The red peak and blue peak (denoted with dashed lines) on the average curve are used to convert pixel location to wavelength.

Finally, the concentration of the targeted chemical can be calculated based on the absorbance result if a calibration equation exists. Alternatively, if the concentration is not known, the analysis result can be used as a new calibration data point. Since the Beer–Lambert Law predicts a linear relationship between absorbance and chemical concentration, a minimum of two calibration data points is required to establish the calibration equation which describes the relationship between absorbance and chemical concentration. When three or more calibration data points are available, the quality of the linear fit may be used to estimate the lower-bound of measurement uncertainty. It is worth pointing out that a separate calibration equation is required for each chemical and each testing reagent. In the iPython Notebook script, all calibration data points are automatically recorded into a calibration file, then at the end of an analysis under user mode, the values of a selected list of process parameters are also recorded into a recipe file.

For an intuitive and wider accessibility of our procedures, the EOSpec application was developed for iOS devices using the Xamarin programming language. It obfuscates the complexity of image analysis and simply outputs the absorbance value to the user.

## Operation instructions

**Fig. 10:**
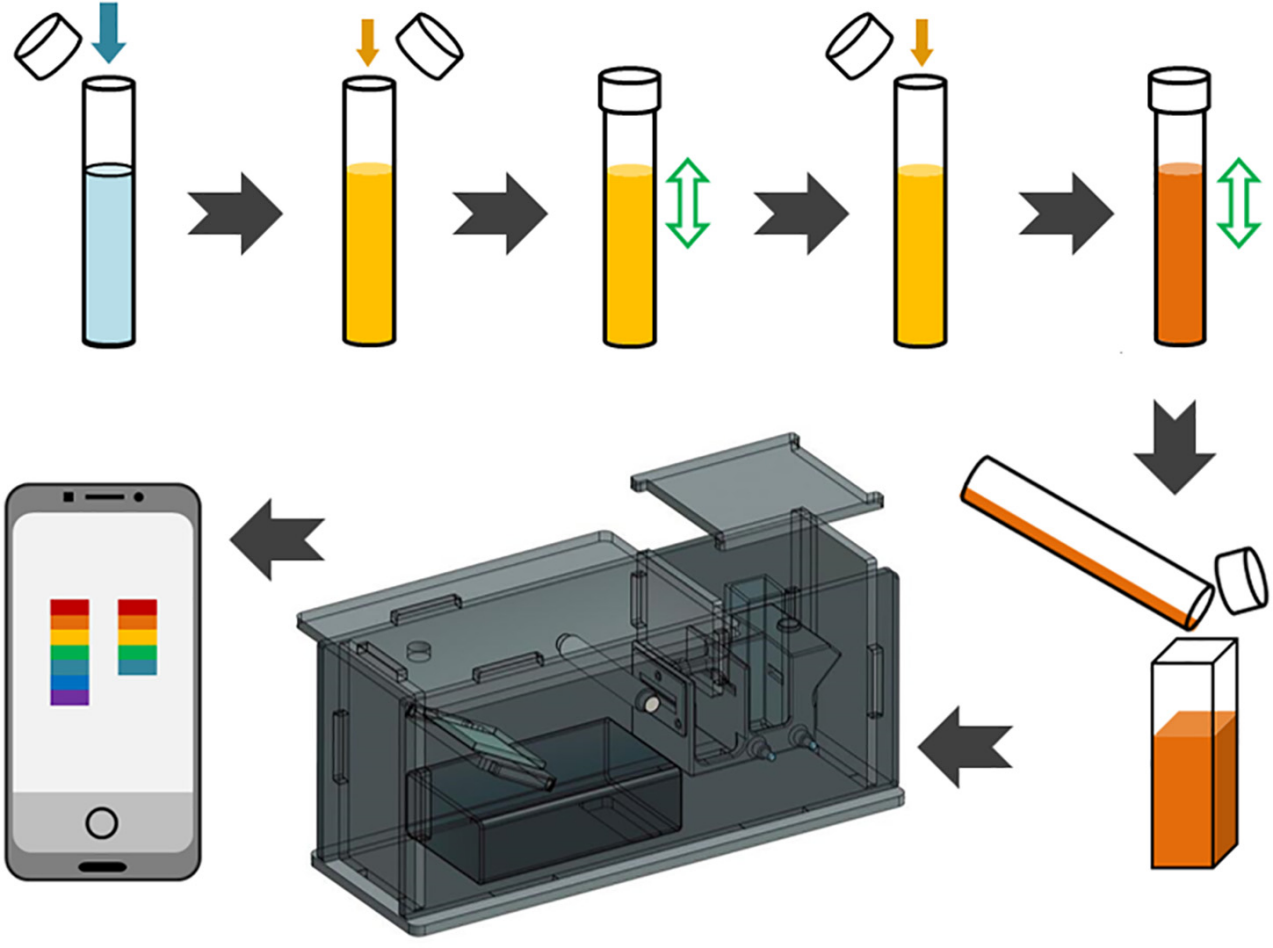
Schematic diagram of the process from water sample to analysis.

The following are brief step-by-step instructions for using the EOSpec to measure absorbance at a given wavelength, a process common to various reagent-based methods of measuring concentration of chemical species. A schematic diagram of operation procedure is shown in Fig. 10.

**Fig. 11:**
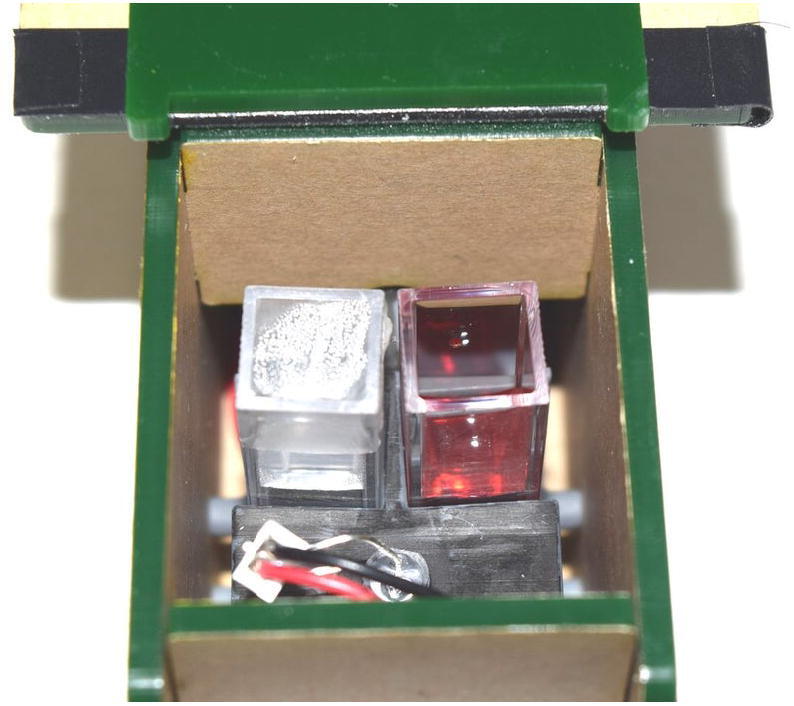
Two cuvettes loaded in holder: Reference cuvette on the left and sample cuvette on the right.

Required are an EOSpec, smartphone, sample to be measured (more than 2.0 ml), testing reagent kit, disposable pipette with volume marks, disposable test tube with lid, and optically clear standard cuvette. When collecting water samples, care should be taken to avoid suspended matters. Generally, it is recommended that samples are collected close to the water surface, because those locations are often more likely to have lower turbidity than the rest of the water body. Use the volume markers on the pipette to transfer the specified amount of sample to the test tube. Choose desired reagent kit based on chemical of interest. Follow the reagent kit’s instruction to ensure consistency. After mixing sample with the reagent, the sample’s color should change depending on compound concentration. For example, for nitrate assay, the resulting color will be yellow, given low nitrate concentration, or it may turn orange to red toward higher concentration. Finally, pour the mixture into sample cuvette. Then place the reference cuvette (usually DI water) into the left-hand-side slot, and the sample cuvette (with colored sample) into the right-hand-side slot, shown in Fig. 11. It is important to ensure the cleanliness of the cuvettes (i.e., free of fingerprints, markings, residues, etc.). When clear or mildly turbid samples are used, it is recommended that the untreated water, without adding any reagent, to be used as reference.

**Fig. 12:**
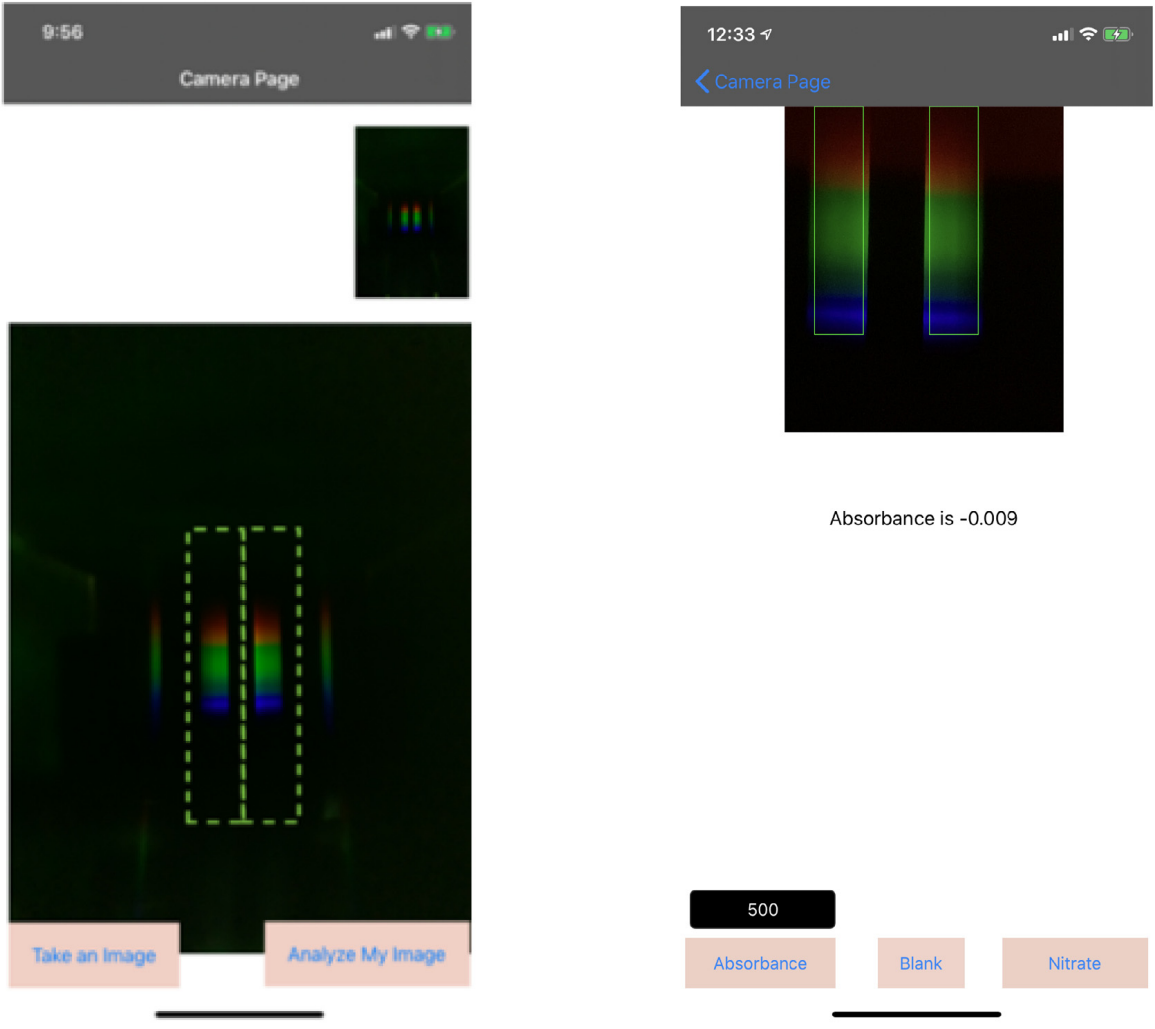
(Left) First screen in the application, where user aligns the spectra into the green box and takes the image. (Right) Second screen in the application. *Blank* button calibrates the device for your phone and surrounding lights for given wavelength written in the box above *Absorbance*. *Nitrate* button measure absorbance of nitrate sample. While *Absorbance* button shows the absorbance for the requested wavelength.

Place the smartphone on top of EOSpec. Open EOSpec application, and align the phone camera with the hole on the top plate. After the two spectra can be seen in the camera viewer, adjust the phone so that the two spectra align with the vertical axis of the green rectangle shown in Fig. 12. Misalignment beyond some point cannot be compensated by the signal finding algorithm.

To calibrate for internal camera settings and left–right baseline intensity differences, put two empty cuvettes into the two slots in the device. Align and take an image, make sure two spectra are oriented correctly - with higher wavelength (red) portions towards the top of the screen, and then click on *Blank*. This step must be performed at the beginning of each series of tests. Then put the reference and the sample cuvettes into their designated slots and take a new image. In the image, the reference spectrum (brighter and complete) should appear on the left, and the sample spectrum (dimmer and maybe with some portions missing) should appear on the right. After taking the desired image, click on *Analyse My Image*, which will advance to the next section of the application in Fig. 12. Enter the desired wavelength for the chemical of interest and click on *Absorbance*. In the current version of the EOSpec application, a *Nitrate* button has been implemented which sets the wavelength to 535 nm, targeting the absorption of the popular API nitrate assay. Other types of tests can be added in the future.

## Validation and characterization

Performance of the EOSpec hardware and associated image analysis was determined by analyzing Nitrate, Sulfide, and Iron (Fe (II)) concentrations in water using absorbance calibration curves. For each chemical species detected, different colorimetric assays were used to react with the target species and produce a color change. This color change can be detected by measuring intensity at specific wavelengths and comparing that value to a reference or blank. These calibration curves represent the change in absorbance as the concentration is adjusted. Similar curves were also constructed for a lab grade spectrometer (Genesys 6 by Thermo-Fisher scientific) and a commercial scale competitor (Vernier Go Direct SpectroVis Plus). These spectrometers were used as standards to compare the EOSpec to when detecting Nitrate, Sulfide, and Iron (Fe (II)). Absorbance data were analyzed using linear regressions ($R^{2}$ values and linear equations) that were present in the calibration curves.

For nitrate, the ‘API Nitrate Test Kit’ for home aquarium nitrate testing was selected and it’s applied method was an acid reduction. This method uses hydrochloric acid to reduce Nitrate to Nitrite and then introduces Sulfanilamide to create an orange-red color which absorbs light at 535 nm [12]. Iron presence in water was determined using a Ferrozine assay. Ferrozine binds to Fe(II) iron in solution and forms a complex that absorbs light most strongly around 550 nm [20]. Sulfide concentrations were determined by the use of a variation on the methylene blue sulfide assay. This method is based on the reaction of sulfide with ferric chloride and dimethyl-p-phenylenediamine to produce methylene blue, which absorbs light most strongly at 660 nm [21].

**Fig. 13:**
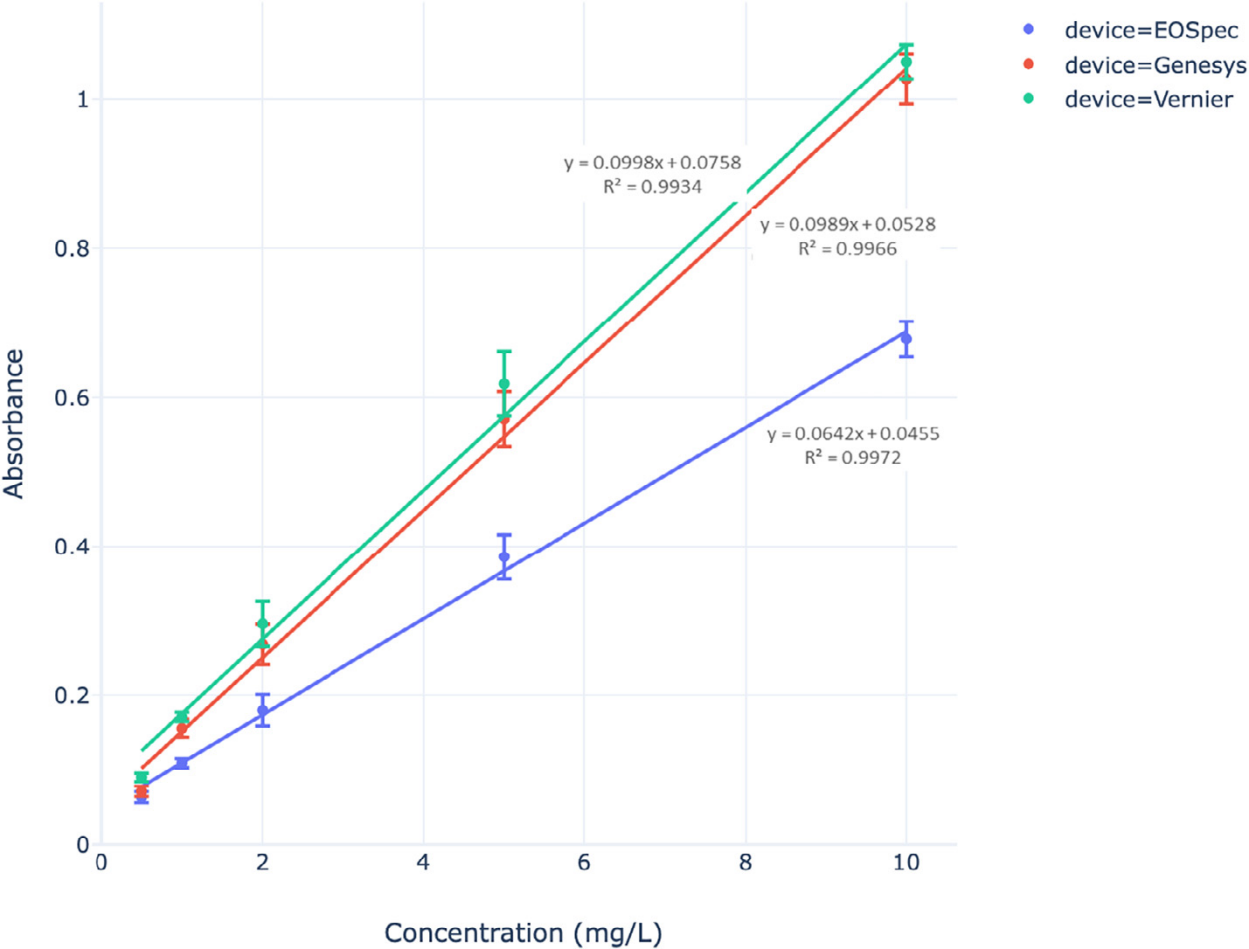
Nitrate Calibration Curve: Average Absorbance for ${\mathrm{NO}}_{3}^{-}$ vs. Concentration using API reagents for each spectrometer.

**Fig. 14:**
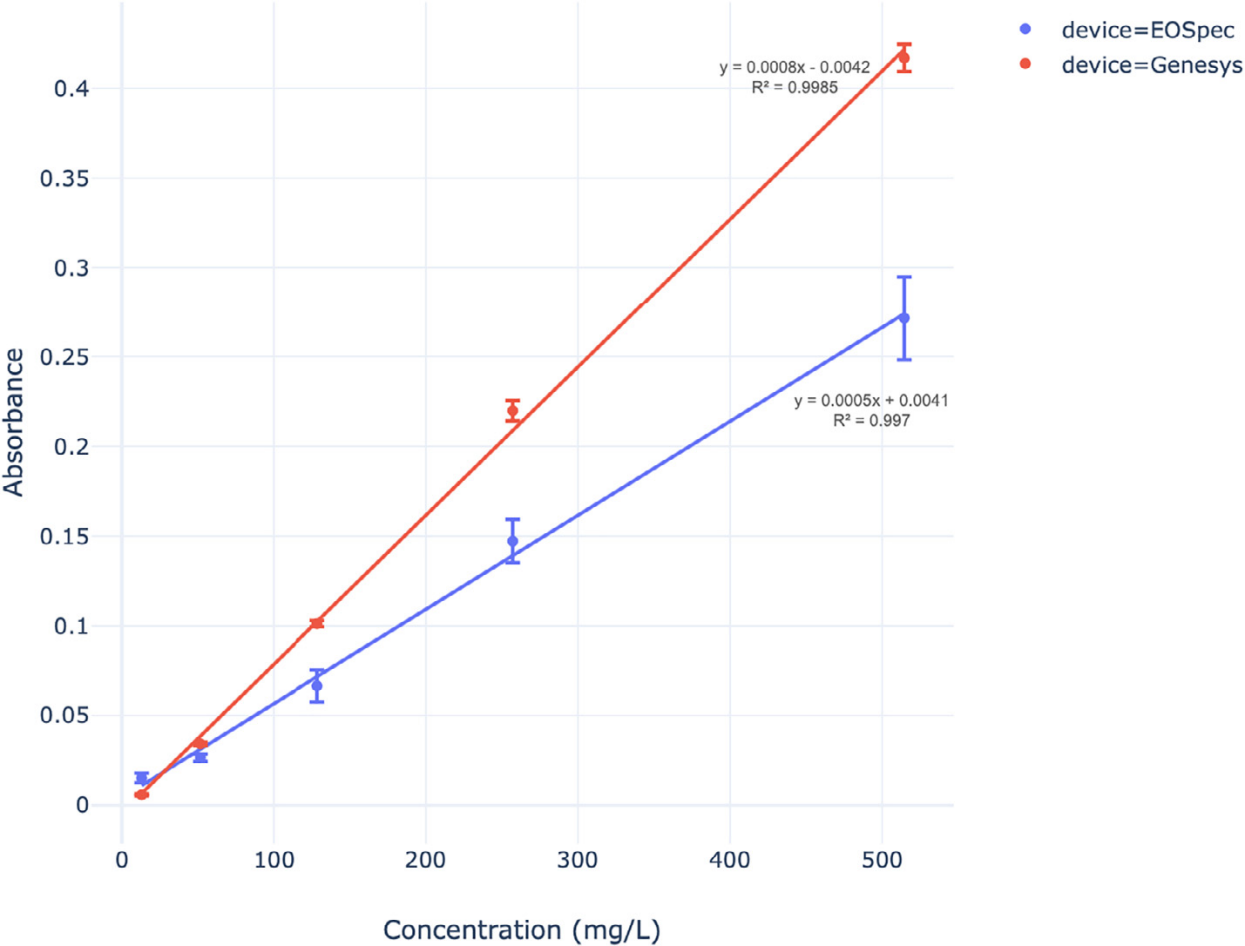
Ferrozine Calibration Curve: Average Absorbance vs. Concentration for Ferrozine with each spectrometer.

**Fig. 15:**
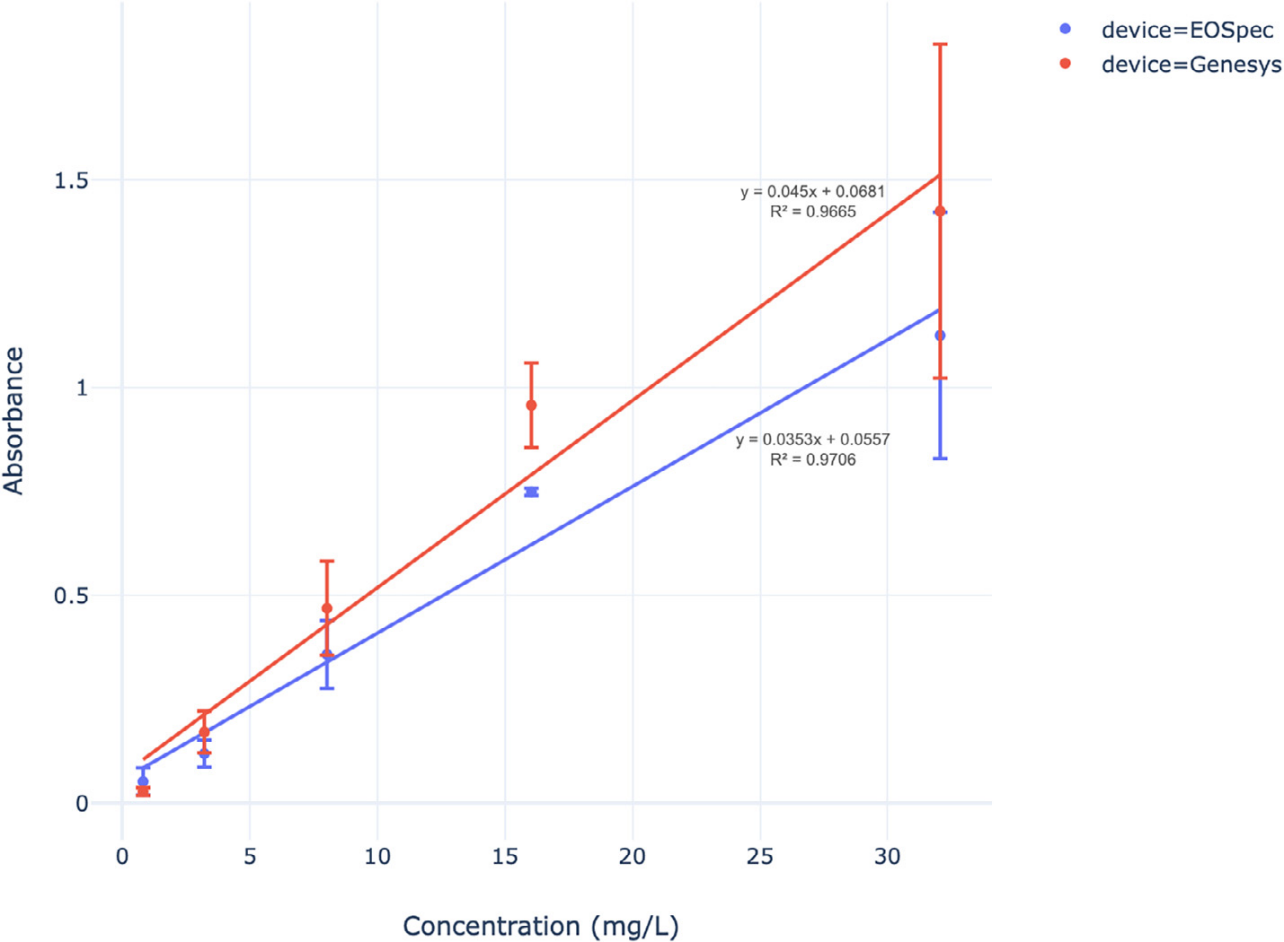
Sulfide Calibration Curve: Average Absorbance vs. Concentration for Sulfide with each spectrometer.

The EOSpec demonstrated (Fig. 13, Fig. 14, Fig. 15) linear correlations ($R^{2}>0.95$) between concentration and absorbance for all 3 of the chemicals tested. This linear relationship is in accordance with the Beer–Lambert law. The performance of the EOSpec is on par with the Vernier and the Genesys 6 in terms of linearity when measuring Nitrate and Sulfide concentrations, indicating that absorbance is changing in a smooth, linear fashion as concentration is adjusted. It can also be observed that the EOSpec calibration curves exhibit lower slopes than both commercial and lab spectrometers, indicating lower sensitivity to change in concentration. Since it is expected that, when the same reagent is used, identical absorbance value should be resulted from samples with the same concentration, the fact that the EOSpec calibration curve deviates from those produced by commercial spectrometers (Fig. 13) suggests the pixel values, *P*, in the digital image may have a power-law dependence on light intensity, *I*, i.e, $P\propto I^{n}$. This nonlinear dependency manifests itself in the change of slope (of the absorbance vs concentration curves) from ∊·l to n·∊·l, and it is found that n≈0.64.

We also measured limits of detection (LoD), which is the smallest concentration reliably distinguished from the blank [22]. LoD for each spectrometer were calculated by setting a baseline (blank) and comparing it to a low concentration of sample. First, a limit of base was calculated using 20 samples of DI water and the following formula: Limit of Base  = Average Blank Sample + (3 x Standard Deviation Blank Sample). Once the limit of base was calculated, the limit of detection was calculated using 20 samples of a low concentration and the following formula: Limit of Detection  = Limit of Base + (3 x Standard Deviation Low Concentration Sample). These equations are based on a 99% confidence interval. 3 multiplied with standard deviation results in a more conservative LoD. EOSpec LoD for ${\mathrm{NO}}_{3}^{-}$ is quite similar to those of the Genesys 6 and the Vernier (Table 3).

**Table 3:** Limits of detection for ${\mathrm{NO}}_{3}^{-}$ on each spectrometer.

**Spectrometer**	**Sample Concentration (mg/L)**	**LOD**
EOSpec	0.6	0.1049
Vernier	0.6	0.1063
Genesys	0.6	0.0927

The EOSpec spectrometer stands as a device that can reliably measure Nitrate, Iron, and Sulfide concentrations using colorimetric assays to measure absorbance. It competes with much more advanced technologies from lab scale (Genesys 6) to commercial scale (Vernier Go Direct SpectroVis Plus) in terms of linearity ($R^{2}$ values), sensitivity (slopes), and the amount of nutrients required to detect a meaningful absorbance value (LOD). The EOSpec can potentially be pushed further in terms of its ability to measure different nutrients (such as Phosphate and Ammonia) by simply changing the reagent selection. This versatility in nutrient selection makes the EOSpec a potential solution to teaching principles of spectrometry in an affordable fashion.

## Declaration of Competing Interest

The authors declare that they have no known competing financial interests or personal relationships that could have appeared to influence the work reported in this paper.
